# Novel insight into the genomic architecture of feed and nitrogen efficiency measured by residual energy intake and nitrogen excretion in growing pigs

**DOI:** 10.1186/1471-2156-14-121

**Published:** 2013-12-20

**Authors:** Mahmoud Shirali, Carol-Anne Duthie, Andrea Doeschl-Wilson, Pieter W Knap, Egbert Kanis, Johan AM van Arendonk, Rainer Roehe

**Affiliations:** 1 Animal and Veterinary Sciences, SRUC, West Mains Road, Edinburgh EH9 3JG, UK; 2 Animal Breeding and Genomics Centre, Wageningen University, P.O. Box 338, 6700 AH Wageningen, The Netherlands; 3 Future Farming Systems, SRUC, West Mains Road, Edinburgh EH9 3JG, UK; 4 Division of Genetics and Genomics, The Roslin Institute, R(D)SVS, University of Edinburgh, Easter Bush, Midlothian EH25 9RG, UK; 5 PIC International Group, Ratsteich 31, 24837 Schleswig, Germany

**Keywords:** Feed efficiency, Growth, Nitrogen excretion, Pigs, Quantitative trait loci, Residual energy intake

## Abstract

**Background:**

Improvement of feed efficiency in pigs is of great economical and environmental interest and contributes to use limited resources efficiently to feed the world population. Genome scans for feed efficiency traits are of importance to reveal the underlying biological causes and increase the rate of genetic gain. The aim of this study was to determine the genomic architecture of feed efficiency measured by residual energy intake (REI), in association with production, feed conversion ratio (FCR) and nitrogen excretion traits through the identification of quantitative trait loci (QTL) at different stages of growth using a three generation full-sib design population which originated from a cross between Pietrain and a commercial dam line.

**Results:**

Six novel QTL for REI were detected explaining 2.7-6.1% of the phenotypic variance in REI. At growth from 60–90 kg body weight (BW), a QTL with a significant dominance effect was identified for REI on SSC14, at a similar location to the QTL for feed intake and nitrogen excretion traits. At growth from 90–120 kg BW, three QTL for REI were detected on SSC2, SSC4 and SSC7 with significant additive, imprinting and additive effects, respectively. These QTL (except for the imprinted QTL) were positionally overlapping with QTL for FCR and nitrogen excretion traits. During final growth (120–140 kg BW), a further QTL for REI was identified on SSC8 with significant additive effect, which overlapped with QTL for nitrogen excretion. During entire analysed growth (60–140 kg BW), a novel additive QTL for REI on SSC4 was observed, with no overlapping with QTL for any other traits considered.

**Conclusions:**

The occurrence of only one overlapping QTL of REI with feed intake suggests that only a small proportion of the variance in REI was explained by change in feed intake, whereas four overlapping QTL of REI with those of nitrogen excretion traits suggests that mostly underlying factors of feed utilisation such as metabolism and protein turnover were the reason for change in REI. Different QTL for REI were identified at different growth stages, indicating that different genes are responsible for efficiency in feed utilisation at different stages of growth.

## Background

Improving feed efficiency, in light of high production costs and environmental impact, is one of the main aims in pig breeding, which contributes to the efficient use of limited resources to feed the world population. Genome scans are of great importance for the identification of genomic regions associated with economically important traits [1]. Additionally, genome scans can reveal the biological background of important traits. Feed conversion ratio (FCR), traditionally used to determine feed efficiency, is inter-related with growth, body composition and feed intake [2].

Residual feed intake (RFI) has become increasingly of interest as an alternative measurement of feed efficiency, which is phenotypically independent from production [3], and has been shown to improve feed efficiency in experimental selection lines [4,5]. Interestingly, among the numerous quantitative trait loci (QTL) mapping studies carried out in pigs, to the best of our knowledge only two studies have performed a genome scan for RFI [6,7] but no study analysed QTL for such trait at different stages of growth. Changes in the energy content of diets during the growing period most often occur, which may have an effect on the accuracy of RFI estimation. Therefore, to obtain more accurate estimates of feed efficiency, in the present study, residual energy intake (REI) was calculated from regressing metabolizable energy intake on estimates of protein and lipid deposition as component traits in successive growth stages. Using the same experimental population as the current study, a large number of QTL for production and feed intake traits have been reported [8-10], suggesting the potential to detect possible QTL for REI in the current population. Shirali et al. [11] identified a substantial favourable phenotypic association between FCR and nitrogen excretions. Therefore, the genomic association between REI and nitrogen excretions can be explored to determine possible methods of mitigating the environmental impact of pig production.

The aims of this study were to detect QTL for REI and nitrogen excretion as measures reflecting feed efficiency and environmental impact, at different stages of growth and over the entire analysed growing period, and to determine the genomic architecture of feed efficiency measured by REI in association with growth, feed intake and nitrogen excretion traits in a commercial population originating from a cross of Pietrain and a commercial dam line.

## Methods

All animal care and handling procedures in the federal testing station were reviewed and approved by the Landwirtschaftskammer Schleswig-Holstein, Rendsburg, Germany.

### Design and data

The QTL mapping analyses was based on animals from a three-generation full-sib design population. The founder generation (F_0_) consisted of 7 unrelated Pietrain grand-sires and 16 unrelated grand-dams bred from a 3-way cross of Leicoma boars with Landrace × Large White dams. All grand-sires were heterozygous (*Nn*) at the *ryanodine receptor 1* (*RYR1*) locus. Of the F_1_ generation, 8 boars and 40 sows were selected to develop the F_2_ generation, which consisted of 315 pigs from the first two parities of the F_1_ sows.

From the F_2_ generation, 48 gilts and 46 barrows were single-housed in straw-bedded pens and fed manually, with feed disappearance recorded on a weekly basis. The remaining 117 gilts and 104 barrows were housed in mixed-sexed groups of up to 15 pigs in straw-bedded pens. Animals housed in groups were fed using electronic feeders (ACEMA 48, ACEMO, Pontivy, France), which recorded feed disappearance at each visit. Pigs started the performance test at about 30 kg body weight and were weighed on a weekly basis. For this study, only the testing period from 60 kg onwards was considered because at this stage pigs were entirely adapted to the electronic feeders. Pigs were weighed at target live weights of 60, 90, 120 and 140 kg, where the average live weight (SD) at these target weights were 61 kg (2.58), 91 kg (2.60), 120 kg (2.69) and 140 kg (2.80), respectively. During growth from 60 to 90 and 90 to 140 kg of body weight, pigs were fed *ad libitum* with a diet containing 13.8 MJ of ME/kg, 17% CP and 1.1% lysine and a diet containing 13.4 MJ of ME/kg, 16.5% CP and 1.0% lysine, respectively. The diets consisted of adequate nutrient supplies to permit maximum protein deposition. For a more detailed description of the data see [12,13].

The deuterium dilution technique was used to determine chemical body composition at each target weight. This technique is an *in vivo* method based on the empty body water content of the pigs [12]. Using this method, the percentage of fat-free substance of pigs was estimated from the empty body water content. Protein and ash content of the empty body were estimated based on the percentage of the fat-free substance. Percentage of lipid content was the deviation of the percentage of fat-free substance from 100%. The accuracy of the deuterium dilution technique to determine body composition has been verified using magnetic resonance imaging on live animals and chemical analysis of serially slaughtered animals using data of the F_1_ population of the present experiment [12,13]. Mohrmann et al. [13] reported the correlations between the estimates for empty body water, fat free substances, and protein in fat-free substances obtained from deuterium dilution technique and chemically analysed methods to be 0.92, 0.90, and 0.85, respectively. Average daily protein (APD) and lipid deposition (ALD) rates were calculated as the difference between protein or lipid content at the two adjacent target weights divided by the number of days between the target weight measurements.

Average daily gain (ADG) was calculated within each growth stage and for the entire analysed growing period (60 to 140 kg). Backfat thickness (BF) was measured on the cold left carcass side. Average daily feed intake (ADFI) was calculated as the sum of feed disappearance (kg) divided by number of days for each stage of growth and over the growing period. Average daily energy intake (ADEI) was calculated as ME content of the diet multiplied by ADFI. The REI was estimated using a regression model for ADEI that included, besides a number of systematic effects, pre-adjusted APD and ALD. The pre-adjustment for APD and ALD was obtained accounting for the same systematic effects as ADEI as described in detail in section statistical analysis. Pre-adjusted traits are used in the estimation model of REI to avoid any influence of systematic effects on the estimates of REI. The FCR was calculated as the sum of feed disappearance (kg) divided by body weight gain (kg) in each stage of growth and the entire analysed growing period.

Three nitrogen excretion traits were estimated at each stage of growth and during the entire analysed growing period: average daily nitrogen excretion (ADNE), nitrogen excretion per body weight gain (NEWG) and total nitrogen excretion (TNE), as an indication of environmental nitrogen pollution by pigs. The ADNE was estimated, using mass balance equation of Whittemore et al. [14], as the difference between average daily nitrogen intake and nitrogen retention, whereby average daily nitrogen retention was obtained as division of APD by 6.25. NEWG was the ratio between ADNE and ADG, and the TNE was calculated as ADNE multiplied by days of growth. More detailed information about the nitrogen excretion traits outlined above is presented in a previous study [11].

### Genotypic data

Blood samples (9 ml) were collected from the F_0_, F_1_ and F_2_ animals by puncture of the vena jugularis and genomic DNA was extracted using the silicagel method following Myakishev et al. [15]. Chromosomes SSC1, SSC2, SSC4, SSC6, SSC7, SSC8, SSC9, SSC10, SSC13 and SSC14 were chosen for genotyping because of their likely associations with lean and fat tissue. All pigs were genotyped for 88 informative microsatellite markers, of which 10, 9, 9, 9, 10, 8, 9, 9, 8 and 7 genomic markers were located on SSC1, SSC2, SSC4, SSC6, SSC7, SSC8, SSC9, SSC10, SSC13 and SSC14, respectively. The position of markers on the genome and their distance from each other, and allele information were obtained using the published USDA linkage map [16]. The average distances between markers were 16.0, 16.5, 16.3, 20.6, 17.3, 18.4, 17.3, 16.0, 18.0 and 17.4 cM and the largest gaps between markers were 27.7, 25.2, 26.5, 28.7, 26.2, 23.1, 21.7, 20.8, 24.0 and 23.6 cM on SSC1, SSC2, SSC4, SSC6, SSC7, SSC8, SSC9, SSC10, SSC13 and SSC14, respectively.

### Statistical analysis

The QTL analysis was performed using GridQTL software [17], which adopts a least squares regression method of QTL mapping [18], and genomic parent-of-origin (imprinting) effect analysis developed by Knott et al. [19]. In this analysis, the estimate of the additive effect is defined as half of the difference between the effects corresponding to pigs homozygous for alleles from the grandpaternal sire line and pigs homozygous for alleles from the grandmaternal dam line. A positive additive genetic value indicates that the allele originating from the grandpaternal sire line (Pietrain) showed a higher effect than the allele from the grandmaternal dam line. The dominance effect is defined as deviation of heterozygous animals from the mean of both types of homozygous animals. A positive dominance value indicates an increase in the trait of interest as a result of a heterozygous genotype. In this study, combined additive and dominance effect analysis was performed for all traits; and in the absence of a significant dominance effect an additive only model was used. Furthermore, all significant QTL were tested for genomic imprinting. In this analysis, imprinting is defined as the phenotypic difference between the two heterozygous states of 2 alleles caused by inheritance of the Pietrain allele from paternal or maternal side. A detectable difference between the two alternative heterozygous states has been used to define the parent of origin effect. A QTL with paternally expressed effect (maternal imprinting) is defined if the effect of parent of origin is in the same direction as the additive effect; whereas, a QTL with maternally expressed effect (paternal imprinting) is defined if the parent of origin and the additive effects are in different directions. Individual QTL analysis was performed for ADFI, ADEI, FCR, APD, ALD, ADG, BF, ADNE, NEWG and TNE using a model that accounted for systematic effects of gender, *RYR1*-genotype, batch, housing type, birth farm, as well as start and end body weight. QTL analysis for REI was performed using a model for ADEI that accounted for, besides the above systematic effects, the pre-adjusted values of APD and ALD. The pre-adjusted measures of APD and ALD were obtained after adjustment for the same systematic effect as described above for the traits using the GLM procedure (SAS Inst. Inc., Cary, NC). The statistical significance threshold level in the QTL analysis was the chromosome-wide significance level obtained by permutation test with 1000 iterations using the GridQTL [17]. Along with investigating each individual growth stage, QTL analyses were performed based on data over the entire analysed growing period from 60 to 140 kg body weight.

## Results

The genome scan identified 47 QTL above the 95% chromosome-wide significance level and 22 QTL at the suggestive level (90% chromosome-wide significance level) at different stages of growth and during the entire analysed growing period for production, feed intake and nitrogen excretion traits (Table 1). The QTL analysis detected 6 QTL for REI, 7 QTL for TNE, 7 QTL for NEWG, 9 QTL for ADNE, 7 QTL for FCR, 6 QTL for ADEI, 10 QTL for ADFI, 6 QTL for APD, 6 QTL for ALD, 4 QTL for average daily gain (ADG) and 1 QTL for BF. Based on the aim of this study, only the QTL associated with feed efficiency and environmental impact due to nitrogen excretion are presented in detail.

**Table 1:** Evidence of QTL for REI, production and nitrogen excretion traits throughout growth

**Growth stage**	**SSC**	**Trait**^**1**^	***F-Ratio***	**Position, cM**	**Marker interval**	**% Var**^**2**^	**Mode of inheritance**	**QTL effect**^**3**^
60 to 90 kg	2	ALD, g/d	4.98^†^	59	SW240-SW1026	3.66	Dominance	−0.02 ± 0.01
	6	ADNE, g/d	5.53^†^	134	SW1881-SW322	3.99	Dominance	3.83 ± 1.16
	6	ADFI, kg/d	5.87*	135	SW1881-SW322	4.07	Dominance	0.18 ± 0.05
	10	ADEI, MJ/d ME	10.61**	44	SW2195	3.79	Additive	−1.12 ± 0.34
	10	ADFI, kg/d	12.88**	44	SW2195	4.44	Additive	−0.10 ± 0.03
	10	ADNE, g/d	11.64**	44	SW2195	4.18	Additive	−2.17 ± 0.64
	14	ADFI, kg/d	6.71**	16	S0089-SW245	4.64	Dominance	−0.18 ± 0.05
	14	ADNE, g/d	7.79**	17	S0089-SW245	5.53	Dominance	−4.18 ± 1.06
	14	ADEI, MJ/d ME	7.22*	18	S0089-SW245	5.11	Dominance	−2.15 ± 0.57
	14	REI, MJ/d ME	8.26**	19	S0089-SW245	6.10	Dominance	−1.75 ± 0.43
90 to 120 kg	1	APD, g/d	7.61*	113	SW1311-SW1828	2.82	Additive	−5.13 ± 1.86
	2	FCR	14.95**	3	SWR2516-SW2623	5.17	Additive	−0.18 ± 0.05
	2	NEWG, g/kg	13.00**	3	SWR2516-SW2623	4.78	Additive	−4.16 ± 1.15
	2	TNE, kg/pig	16.11**	4	SWR2516-SW2623	4.02	Additive	−0.13 ± 0.03
	2	REI, MJ/d ME	9.18*	16	SW2623-SWR783	3.46	Additive	−0.96 ± 0.32
	4	TNE, kg/pig	10.39*	14	SW489-S0301	3.81	Additive	0.10 ± 0.03
	4	FCR	12.67**	17	SW489-S0301	4.42	Additive	0.16 ± 0.04
	4	NEWG, g/kg	8.57*	19	SW489-S0301	3.20	Additive	3.33 ± 1.14
	4	ADG, g/d	10.39*	24	SW489-S0301	3.67	Additive	−37.54 ± 11.65
	4	REI, MJ/d ME	5.05*	130	MP77-SW856	5.63	Imprinting	0.60 ± 0.26
	6	ADNE, g/d	8.51*	70	S0087-SW122	3.15	Additive	−2.00 ± 0.68
	6	FCR, MJ/d ME	8.32*	105	S0228	2.95	Additive	−0.13 ± 0.04
	6	ADFI, kg/d	4.76^†^	128	SW1881-SW322	3.37	Dominance	0.15 ± 0.05
	7	ADNE, g/d	5.40*	58	SW1841-S0087	3.97	Dominance	−3.50 ± 1.07
	7	TNE, kg/pig	4.83^†^	88	SW122-S0228	3.57	Dominance	−0.17 ± 0.06
	7	NEWG, g/kg	4.88^†^	116	SWR773	3.65	Dominance	−5.27 ± 1.93
	7	REI, MJ/d ME	7.77*	117	SWR773	2.94	Additive	0.75 ± 0.27
	7	FCR	5.29^†^	117	SWR773	3.73	Dominance	−0.22 ± 0.08
	10	ADG, g/d	9.09*	3	SW830-SWR136	3.22	Additive	−34.19 ± 11.34
	10	ADFI, kg/d	7.51*	6	SW830-SWR136	2.67	Additive	−0.08 ± 0.030
	13	NEWG, g/kg	9.06*	119	SW2440-S0291	3.38	Additive	3.43 ± 1.14
120 to 140 kg	1	ADG, g/d	4.5^†^	116	SW1311-SW1828	3.20	Dominance	60.73 ± 20.32
	2	ADFI, kg/d	5.63*	82	SW1370-SWR2157	3.96	Dominance	0.20 ± 0.06
	2	TNE, kg/pig	8.45*	115	SWR345	3.19	Additive	0.08 ± 0.03
	2	NEWG, g/kg	9.84*	116	SWR345	3.73	Additive	4.53 ± 1.45
	4	ALD, g/d	7.43**	5	SW2404-SW489	5.57	Dominance	−38.44 ± 12.34
	6	ADFI, kg/d	5.00^†^	148	SW322	3.53	Dominance	0.19 ± 0.06
	6	ADEI, MJ/d ME	4.61^†^	150	SW322	3.32	Dominance	2.10 ± 0.69
	7	ALD, g/d	4.81^†^	28	SWR1343-SW2155	3.68	Dominance	49.33 ± 15.93
	8	REI, MJ/d ME	14.46**	0	SW2410	5.53	Additive	−1.26 ± 0.33
	8	ADNE, g/d	11.12*	0	SW2410	4.16	Additive	−2.56 ± 0.77
	9	ADG, g/d	5.11^†^	81	S0019-SW2093	3.62	Dominance	70.29 ± 22.74
	9	ADEI, MJ/d ME	6.88*	82	S0019-SW2093	4.88	Dominance	2.56 ± 0.73
	9	ADFI, kg/d	5.48*	82	S0019-SW2093	3.86	Dominance	0.20 ± 0.06
	9	APD, g/d	4.47^†^	86	S0019-SW2093	3.43	Dominance	10.76 ± 3.67
60 to 140 kg	2	APD, g/d	6.44^†^	0	SWR2516-SW2623	2.51	Additive	3.37 ± 1.33
	2	FCR	12.08**	4	SWR2516-SW2623	4.09	Additive	−0.09 ± 0.03
	2	TNE, kg/pig	9.91*	4	SWR2516-SW2623	3.57	Additive	−0.18 ± 0.06
	2	NEWG, g/kg	8.81*	13	SW2623-SWR783	3.50	Additive	−2.22 ± 0.75
	4	NEWG, g/kg	8.08*	15	SW489-S0301	3.22	Additive	1.97 ± 0.69
	4	TNE, kg/pig	8.77*	22	SW489-S0301	3.17	Additive	0.16 ± 0.06
	4	FCR	7.54^†^	24	SW489-S0301	2.60	Additive	0.07 ± 0.02
	4	REI, MJ/d ME	7.16^†^	130	MP77-SW856	2.70	Additive	−0.45 ± 0.17
	6	ALD, g/d	4.91^†^	131	SW1881-SW322	3.58	Dominance	20.15 ± 6.45
	6	ADEI, MJ/d ME	7.56**	133	SW1881-SW322	5.51	Dominance	2.22 ± 0.57
	6	APD, g/d	4.91^†^	134	SW1881-SW322	3.64	Dominance	7.43 ± 2.41
	6	ADNE, g/d	7.28*	134	SW1881-SW322	5.17	Dominance	3.60 ± 0.95
	6	ADFI, kg/d	7.50*	135	SW1881-SW322	4.92	Dominance	0.18 ± 0.05
	6	BF, cm	11.42*	163	SW322-SW2052	3.75	Additive	−0.12 ± 0.04
	7	APD, g/d	4.69^†^	5	SW2564-SWR1343	3.42	Dominance	4.81 ± 2.13
	7	TNE, kg/pig	6.53*	111	SW632-SWR773	4.66	Dominance	−0.31 ± 0.10
	8	APD, g/d	6.90*	101	SW374-SW1551	2.51	Additive	3.54 ± 1.35
	9	ALD, g/d	4.58^†^	18	SW21-SW911	3.32	Dominance	20.64 ± 6.96
	9	ADEI, KJ/d ME	5.66*	82	S0019	4.19	Dominance	1.59 ± 0.48
	9	ADNE, g/d	5.23*	82	S0019	3.77	Dominance	2.55 ± 0.79
	14	ALD, g/d	4.31^†^	14	S0089-SW245	3.14	Dominance	−13.34 ± 6.25
	14	ADNE, g/d	4.56^†^	14	S0089-SW245	3.30	Dominance	−2.65 ± 0.89
	14	ADFI, kg/d	5.13*	15	S0089-SW245	3.41	Dominance	−0.14 ± 0.04
	14	FCR	4.55^†^	88	SW1557-SWC27	3.13	Dominance	0.15 ± 0.05

^†^= 0.1, * = 0.05 and ** = 0.01 chromosome-wide significance level.

^1^REI = residual energy intake; ADEI = average daily energy intake; APD = average protein deposition; ALD = average lipid deposition; FCR = feed conversion ratio; TNE = total nitrogen excretion; ADNE = average daily nitrogen excretion; NEWG = nitrogen excretion per weight gain; BF = backfat thickness.

^2^Percentage of variance explained by the QTL calculated as the proportion of residual sum of square from the full model divided by the residual sum of square from null model (excluding QTL effect).

^3^Estimated additive, dominance or imprinting effects and their standard error.

### QTL for REI during growth from 60 to 90 kg

**Figure 1:**
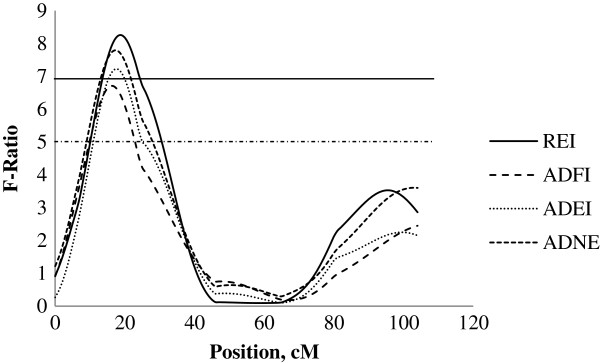
**Evidence of QTL for performance traits on SSC14 during 60–90 kg BW growth stage.** Test-statistic along SSC14 for evidence of QTL for residual energy intake (REI), average daily feed intake (ADFI), average daily energy intake (ADEI) and average daily nitrogen excretion (ADNE) at the growth period of 60 to 90 kg body weight. The solid and dashed horizontal lines denote the 99% and 95% chromosome-wide significance level, respectively.

On SSC14, a QTL with significant dominance effects was identified for REI at position 19 centimorgan (cM) close to SW245 with a QTL effect of −1.75 ± 0.43 MJ/d ME, explaining 6.1% of the phenotypic variation in REI. This QTL was located in a position close to QTL for ADNE (−4.18 ± 1.06, g/d), ADEI (−2.15 ± 0.57, MJ/d ME) and ADFI (−0.18 ± 0.05, kg/d) (Figure 1). No further QTL for production traits (e.g. ADG, APD and ALD) were identified in this chromosomal region, indicating that the QTL for REI in this region is independent to production traits.

### QTL for REI during growth from 90 to 120 kg

**Figure 2:**
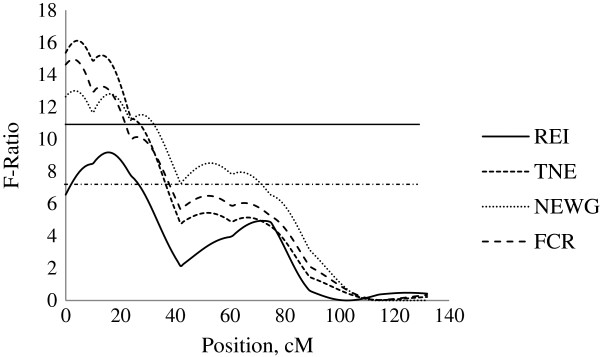
**Evidence of QTL for performance traits on SSC2 during 90–120 kg BW growth stage.** Test-statistic along SSC2 for evidence of QTL for residual energy intake (REI), total nitrogen excretion (TNE), nitrogen excretion per weight gain (NEWG) and feed conversion ratio (FCR) at the growth from 90 to 120 kg body weight. The solid and dashed horizontal lines denote the 99% and 95% chromosome-wide significance level, respectively.

**Figure 3:**
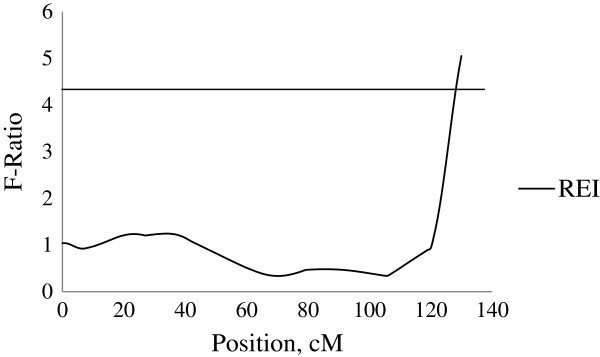
**Evidence of QTL for performance traits on SSC4 during 90–120 kg BW growth stage.** Test-statistic along SSC4 for evidence of QTL for residual energy intake (REI) at the growth period of 90 to 120 kg body weight. The solid horizontal line denotes the 95% chromosome-wide significance level.

**Figure 4:**
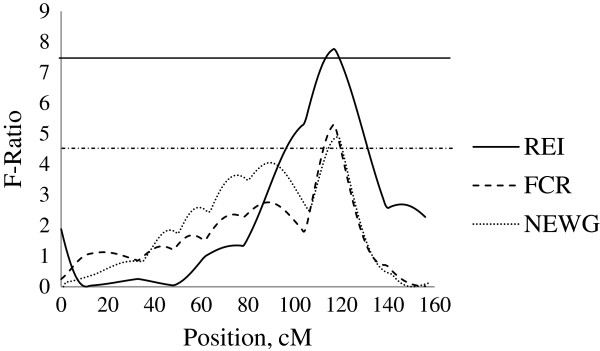
**Evidence of QTL for performance traits on SSC7 during 90–120 kg BW growth stage.** Test-statistic along SSC7 for evidence of QTL for residual energy intake (REI), feed conversion ratio (FCR) and nitrogen excretion per weight gain (NEWG) at the growth period of 90 to 120 kg body weight. The solid horizontal line denotes the 95% chromosome-wide significance level for additive QTL for REI, and the dashed horizontal line denotes the 90% chromosome-wide significance level for dominance QTL for FCR and NEWG.

Three QTL for REI were detected at 90 to 120 kg body weight. Firstly, on SSC2 at 16 cM close to SWR783, an additive QTL was detected with a significant QTL effect of −0.96 ± 0.32 MJ/d ME, explaining 3.5% of phenotypic variance in REI. This QTL was identified at the similar location as the QTL for FCR (−0.18 ± 0.05), TNE (−0.13 ± 0.03, kg/pig) and NEWG (−4.16 ± 1.15, g/kg) (Figure 2). Secondly, on SSC4 a unique QTL for REI was captured which showed paternal genomic imprinting (i.e. only the maternally inherited allele is expressed). This QTL was identified at position 130 cM close to SW856 with a QTL effect of 0.60 ± 0.26 MJ/d ME, explaining 5.6% of the phenotypic variance in REI (Figure 3). No further QTL were identified in this chromosomal region for any other traits in this study. Thirdly, on SSC7, an additive QTL was identified at position 117 cM close to SWR773 with a significant QTL effect of 0.75 ± 0.27 MJ/d ME, explaining 2.9% of the phenotypic variance in REI. This QTL was identified at the similar location as a dominant QTL for FCR (−0.22 ± 0.08) and NEWG (−5.27 ± 1.93, g/kg) (Figure 4).

### QTL for REI during growth from 120 to 140 kg

**Figure 5:**
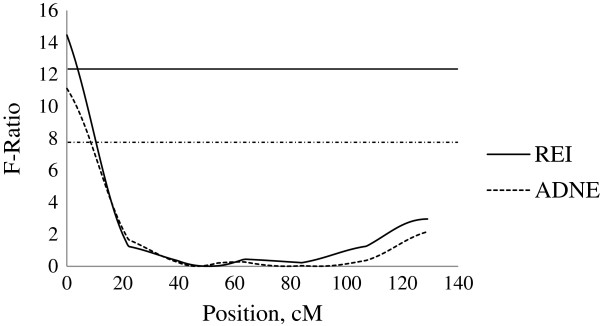
**Evidence of QTL for performance traits on SSC8 during 120–140 kg BW growth stage.** Test-statistic along SSC8 for evidence of QTL for residual energy intake (REI) and average daily nitrogen excretion (ADNE) at the growth period of 120 to 140 kg body weight. The solid and dashed horizontal lines denote the 99% and 95% chromosome-wide significance level, respectively.

On SSC8, an additive QTL for REI at 120 to 140 kg body weight was detected at position 0 cM close to SW2410 with a QTL effect of −1.26 ± 0.33 MJ/d ME, explaining 5.5% of the phenotypic variance in REI. Within this chromosomal region, a QTL for ANDE was also located, with an additive mode of inheritance associated with a reduction in ADNE (−2.56 ± 0.77, g/d) (Figure 5).

### QTL for REI during the entire analysed growth period (60 to 140 kg)

**Figure 6:**
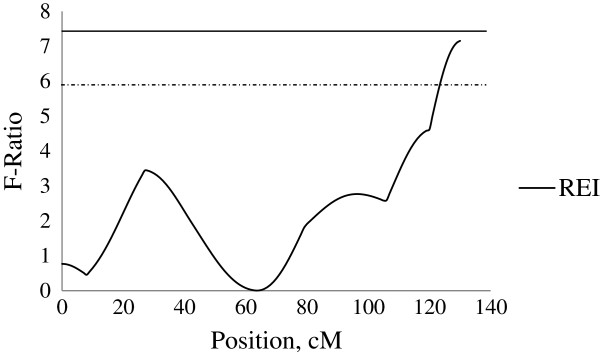
**Evidence of QTL for performance traits on SSC4 during 60–140 kg BW growth stage.** Figure 6. Test-statistic along SSC4 for evidence of QTL for residual energy intake (REI) for the entire analysed growth period (60 to 140 kg body weight). The solid and dashed horizontal lines denote the 95% and 90% chromosome-wide significance level, respectively.

When investigating the entire analysed growing period (60 to 140 kg) none of the above QTL were identified. An additional QTL was however identified on SSC4 at 130 cM for REI in 90% significance level with additive effects accounting for 2.7% of the phenotypic variance in REI. This chromosomal region did not harbour any QTL for the other traits analysed in this study (Figure 6).

### QTL for nitrogen excretions during growth from 60 to 90 kg

In total 23 QTL were identified throughout the genome for traits associated with nitrogen excretions. Of these QTL, 15 were identified at specific stages of growth and 8 were only identified when considering the entire analysed growing period. On SSC6, a dominance QTL for ADNE (3.83 ± 1.16, g/d) was identified at positions 134 cM, between markers SW1881 and SW322, which was in a similar region to a QTL for ADFI with significant dominance genetic effects (0.18 ± 0.05, kg/d). On SSC10, at position 44 cM a significant QTL was identified for ADNE with an additive genetic effect indicating that the grandpaternal Pietrain allele is favourably associated with a reduction in ADNE by −2.17 ± 0.64 g/d. In addition, QTL were identified within this region of SSC10 for ADEI and ADFI with significant additive effects of −1.12 ± 0.34 MJ/d ME and −0.10 ± 0.03 kg/d, respectively.

### QTL for nitrogen excretions during growth from 90 to 120 kg

On SSC4, at 14 to 24 cM, between SW2404 and SW489, QTL were identified for ADG, NEWG, TNE and FCR with significant additive genetic effects. This genomic region was associated with an unfavourable effect of the Pietrain allele on reduction of ADG (−37.54 ± 11.65, g/d) combined with an unfavourable increase in NEWG (3.33 ± 1.14, g/kg), TNE (0.10 ± 0.03, kg/pig) and FCR (0.16 ± 0.04). On SSC6, three QTL were identified at three different positions for ADNE, FCR and ADFI at 70, 105 and 128 cM, respectively. On SSC7, QTL were identified at positions 88, 58 and 117 cM for TNE, ADNE and NEWG, respectively, indicating the association of different regions of this chromosome with possible environmental pollution of pig production. On SSC13, a unique additive QTL for NEWG was identified at position 119 cM, between markers SW2440 and S0291, which explained 3.4% of the phenotypic variation. This QTL showed that the additive genetic effect of the allele originating from the Pietrain grandpaternal breed was associated with an increase in nitrogen excretions by 3.43 ± 1.14 g/kg of body weight.

### QTL for nitrogen excretions during growth from 120 to 140 kg

On SSC2, QTL at positions 115 to 116 cM, close to SWR345, identified with the Pietrain allele showing an unfavourable additive genetic association with TNE (0.08 ± 0.03, kg/pig) and NEWG (4.53 ± 1.45, g/kg). On SSC6, at positions 148 and 150 cM, close to SW322, 2 QTL with a significant dominance effect were identified for ADFI and ADEI, respectively. This region also showed QTL association with ADFI at other stages of growth.

### QTL for nitrogen excretions during the entire analysed growing period (60 to 140 kg)

On SSC2, at positions 0 to 4 cM, between SWR2516 and SW2623, favourable additive genetic effects of the Pietrain allele with APD, FCR and TNE were identified, where the grandpaternal Pietrain breed was associated with an increase in APD (3.37 ± 1.33, g/d), a reduction in FCR (−0.09 ± 0.03) and TNE (−0.18 ± 0.06, kg/pig). In addition, at a slightly different location on SSC2 (13 cM), close to marker SW2623, a favourable additive genetic effect of the Pietrain allele for NEWG (−2.22 ± 0.75, g/kg) was identified, indicating that these regions on the same chromosome are highly associated with both feed efficiency and potentially environmental pollution. On SSC4, at positions 15 to 24 cM, between SW489 and S0301, positive additive genetic associations with FCR (0.07 ± 0.02), NEWG (1.97 ± 0.69, g/kg) and TNE (0.16 ± 0.06, kg/pig) were identified. On SSC6 from 131 to 135 cM, between SW1881 and SW322, 5 QTL with significant dominance effects were identified suggesting that the heterozygous genotype is associated with an unfavourable increase in ADNE (3.60 ± 0.95, g/d) and ALD (20.15 ± 6.45, g/d) as well as a favourable increase in APD (7.43 ± 2.41, g/d) related to an increased ADEI (2.22 ± 0.57, MJ/d ME) and ADFI (0.18 ± 0.05, kg/d). In addition, a favourable additive QTL effect of the Pietrain allele was identified for BF (−0.12 ± 0.04) around this region. On SSC7, a QTL with significant dominance effects for TNE was identified at 111 cM, between SW632 and SWR773, with a favourable effect of −0.31 ± 0.10, kg/pig. This region did not show any association with any other traits in this study. On SSC9, position 82 cM had a significant dominance association with ADNE (2.55 ± 0.79, g/d) and ADEI (1.59 ± 0.48, MJ/d ME) without any influence on production traits, indicating that a heterozygous genotype was associated with an unfavourable increase in these traits. On SSC14, three QTL with significant dominance effects were identified between positions 14 and 15 cM, between S0089 and SW245, with favourable decrease in ADNE (−2.65 ± 0.89, g/d) and ALD (−13.34 ± 6.25, g/d) related to a decrease in ADFI (−0.14 ± 0.04, kg/d).

## Discussion

### QTL for REI

**Figure 7:**
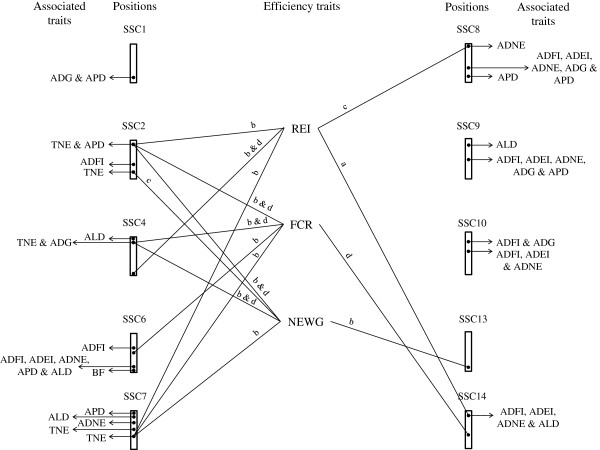
**QTL profile of performance traits throughout genome.** The genomic architecture of feed and nitrogen efficiency in association with growth, feed intake and nitrogen excretion traits are presented using QTL information for residual energy intake (REI), feed conversion ratio (FCR), nitrogen excretion per weight gain (NEWG), total nitrogen excretion (TNE), average daily nitrogen excretion (ADNE), average daily feed intake (ADFI), average daily energy intake (ADEI), average daily gain (ADG), average daily protein deposition (APD) and average daily lipid deposition (ALD). The a, b, c and d represent 60 to 90 kg, 90 to 120 kg, 120 to 140 kg growth stages and the entire analysed growth period (60 to 140 kg), respectively.

In the current study, six novel QTL were detected for REI at different stages of growth, bringing new insight into the genomic architecture of efficiency of feed utilisation. Based on QTL information, the genomic architecture of feed and nitrogen efficiency in growing pigs is broadly illustrated in Figure 7. The identification of QTL for REI in this study in different genomic locations for different stages if growth, suggests that different genes are switched on and off throughout growth.

At early stage of growth (60 to 90 kg body weight), the identified QTL for REI on SSC14 expressed dominance effects and was overlapping with QTL for both ADNE and ADEI. Furthermore, the animals with heterozygous genotype had reduced lipid deposition, feed intake and nitrogen excretion during the entire analysed growing period. In the current study, this QTL region (S0089-SW245) did not show any association with protein growth indicating that this chromosomal region of SSC14 harbours a QTL for REI which is caused by reduced feed intake. The reduced feed intake may again be the reason for the identified QTL for ALD. However, it cannot be distinguished from this study whether low feed intake is the driver for low lipid deposition or vice versa. Moreover, it is difficult to determine whether this region carries one or multiple QTL for those traits. De Koning et al. [19] reported a maternally imprinted QTL for backfat (measured by ultrasound) in this region (SW857-SW295) for a cross between Chinese Meishan and Dutch commercial pigs. Additionally, Rohrer and Keele [20] reported a suggestive additive QTL for average backfat thickness in this region (SW510-SW2439) for a three generation reciprocal backcross of Meishan and Large White composite pigs. Furthermore, a QTL for daily feed intake has been reported in the same region (SW857-S0007) by Liu et al. [21]. The result suggests that the QTL for REI in this region of SSC14 may be associated with improved production efficiency and reduced nitrogen excretions through reduction in energy usage and fatness. Furthermore, Sahana et al. [22] reported eight SNPs to be associated with FCR on SSC14 in different genomic regions (120 to 124 cM) to the QTL obtained in current study for FCR and REI. Fan et al. [6] suggested an association of TCF7L2 c.646 + 514A > G SNP on SSC14, at 134.5 cM to 134.78 cM position, with RFI as possible genetic markers for this trait. Fan et al. [6] concluded that the involvement of these SNPs with variation in RFI suggests that there is a common pathway or network regulating fatness, energy balance and feed intake.

At 90 to 120 kg of growth, a QTL for REI on SSC2 showed associations with QTL for TNE, NEWG and FCR. Additionally, this region was found to have an association with protein deposition during the entire analysed growing period, suggesting that the allele originating from the Pietrain breed is associated with an increase in protein deposition and an improvement in feed efficiency and reduced environmental pollution of pig production. The same QTL for FCR using the same experimental population as in the current study was reported in a previous study where the authors also suggested the segregation of the IGF2 allele as a candidate gene for this QTL, which is associated with fatness and growth [8]. In addition, this region has been shown in the literature to be associated with ADG, ADFI, body weight, ultrasonic backfat and FCR [7,8,19,23-25]. The QTL for REI, FCR, NEWG, TNE and APD reported in the current study had an additive mode of inheritance compared to the QTL for IGF2, which showed genomic imprinting, indicating that, besides the QTL for IGF2, there might be an additional QTL for APD on SSC2 around this location causing the improvement in efficiency of protein deposition. The results described in this study, indicate that this chromosomal region may play a role in improving the efficiency of feed utilisation through an increase in leanness, decrease in feed conversion ratio and consequently a reduction in the environmental impact of pig production.

During growth from 90 to 120 kg body weight, the QTL for REI on SSC4 showed paternal imprinting so that the maternally inherited Pietrain allele expressed an undesirable increase in REI. This QTL showed a change in mode of inheritance depending on the considered growth period. At growth from 60 to 140 kg body weight, this QTL had additive effect with a desirable reduction in REI. This may indicate that the responsible gene (or genes) in this region have changed in function during the growing period. It has to be considered that the estimated parent of origin effects may not caused by imprinting but maternal effects as this effect can be confounded with imprinting effects as estimated in this study. However, a maternal effect would most likely be expected at earlier (60 to 90 kg) and not at later stage of growth (90 to 120 kg). There are reports of QTL around this region for ADG, loin muscle area and ultrasonic backfat [26,27]. This chromosome has been shown to harbour a QTL for FCR with an additive mode of inheritance in a region different to the QTL for REI, as reported by Duthie et al. [8], using the same experimental population as in the current study. In addition, Sahana et al. [22] reported two significant SNP associated with FCR on SSC4 but at different positions (63.9 cM and 64.0 cM, respectively) to the QTL identified in the present study for FCR and REI. The lack of association with feed intake traits suggest that the REI QTL may be associated with underlying causes of variation in REI such as metabolism, protein turnover, etc.

At 90 to 120 kg of body weight growth, the identified additive QTL for FCR on SSC6 was the only QTL associated with feed efficiency on this chromosome in the current study. In agreement, Yue et al. [28] reported a QTL for FCR around the FCR QTL region identified in the current study. Fan et al. [6] suggested an association of fat mass and obesity related p.Ala198Ala SNP on SSC6, at 28.28 cM to 28.33 cM. In the current study, in this chromosomal region, no QTL for REI was detected, which could be expected as REI is adjusted for fat growth, assuming the QTL for fatness is the underlying biological reason for the FCR QTL.

At 90 to 120 kg of growth, the identified additive QTL for REI on SSC7 (between SW632 and SWR773) was overlapping with the dominant QTL for FCR and NEWG. In addition, the QTL of this genomic region were associated with a dominance QTL effect for TNE during the entire analysed growing period. The results suggest that heterozygous genotypes at this QTL are associated with efficient use of energy and nitrogen intake during growth. In the present population, when the allele originating from the Pietrain grandpaternal breed is present, an increase in REI was obtained. This may be caused by one QTL with pleiotropic effects or by multiple QTL with different mode of inheritance in this region. In contrast to our study, this region has been shown to be associated with ADG, average feeding rate and backfat thickness [23,25,29,30]. Furthermore, Zhang et al. [31] reported a FCR QTL on this chromosome at 64.8 cM, from a cross of White Duroc and Chinese Erhualian breeds, whereas this QTL was not detected in the current study.

At the latest growth period considered in this study (120 to 140 kg), the QTL for REI on SSC8 (between SW2410 and SW905) was only found to have an association with nitrogen excretions. These results suggest that the QTL for REI may be associated with underlying variation in efficiency of digestion, feed utilisation, protein turnover, etc., due to independence of REI from production, and no positional association with QTL for feed intake traits in this region. However, other studies have found QTL for ADG in this region (e.g. [23,31,32]).

Following analyses for epistatic QTL in the current study, no epistatic QTL was detected for REI at any of the growth stages considered in this study.

### QTL for nitrogen excretions

During early stage of growth (60 to 90 kg), the QTL region on SSC6 expressing dominance effect association with ADFI and ADNE also was found to have a dominance effect associated with increase in ADFI, ADEI and ADNE at different stages of growth. Furthermore, this region was also associated with an increase in APD and ALD during the entire analysed growing period from 60 to 140 kg body weight. This suggests that there might be a pleiotropic QTL or QTL in high linkage disequilibrium that increases production such as APD and to a higher extent ALD, therefore, resulting in increased feed intake, energy usage and consequently nitrogen excretions. These QTL for ADFI at 60 to 90 kg, and 90 to 120 kg growth stages has been reported by Mohrmann et al. [10] using the same experimental population as in the current study. Gilbert et al. [7] reported a QTL for FCR in this region for Pietrain-Large White backcross. In addition, at 90 to 120 kg of growth, on SSC6, the additive effect QTL associated with reduction of ADNE indicates that the allele originating from the Pietrain grandpaternal breed is associated with a reduction in nitrogen excretions. This region was not associated with any other trait analysed in this study suggesting that this is a unique QTL for nitrogen efficiency. However, Gilbert et al. [7] reported a QTL for ADFI around this region (83 cM), which would suggests that the increase in ADNE may be due to an increase in feed intake as these traits are highly correlated [11].

At the 60 to 90 kg stage of growth, the QTL on SSC10 associated with ADNE, ADFI and ADEI indicates that the allele originating from the Pietrain grandsire is associated with reduced feed intake, energy usage, and consequently may results in a reduction in the environmental impact of pig production. The QTL for ADFI has been previously reported in a study which utilised the same experimental data [8]. A QTL for ADG in nearby region has been previously reported for a cross of outbred Wild boar and Large White pigs [23]. Furthermore, during growth from 90 to 120 kg, the Pietrain allele at the QTL on SSC10, between markers SW830 and SWR136, had an unfavourable additive genetic association with ADFI (−0.08 ± 0.03, kg/d) and ADG (−34.19 ± 11.34, g/d). Duthie et al. [8] reported a QTL with unfavourable additive effects of the Pietrain on APD in this region at the same growth period, using the same experimental data as in the current study. This suggests that the allele originating from Pietrain grandsire breed is associated with a reduction in protein deposition, and consequently growth and the feed intake required for growth.

At 90 to 120 kg body weight, the QTL region on SSC4 between SW489 and S0301 had an additive effect of the Pietrain allele associated with a reduction in ADG, and an increase in NEWG, TNE and FCR. In addition, this region showed the same unfavourable QTL effects of the Pietrain allele on FCR, NEWG and TNE during the entire analysed growing period. Duthie et al. [8] reported this QTL for FCR using the same experimental data as in the current study. These results indicate that the additive allele from the grandpaternal Pietrain breed is associated with a reduction in production, and consequently an increase in FCR and nitrogen excretion. This is surprising as the Pietrain breed has been greatly selected for improved productivity.

During 90 to 120 kg of growth, the favourable dominance QTL for ADNE on SSC7 (58 cM) and TNE (88 cM) suggest that heterozygous animals have less nitrogen excretions. These regions were not associated with any other traits in the current study, suggesting a unique QTL for nitrogen efficiency. However, Gilbert et al. [7] reported a suggestive (*P* < 0.1) QTL for ADFI at the same position as the QTL for ADNE and additionally a QTL for FCR (74 cM) in nearby region to the QTL for TNE.

At 90 to 120 kg of growth, the additive QTL on SSC13 associated with an increase in NEWG showed no association with any growth and feed efficiency traits in the current study. This indicates that the allele originating from the Pietrain grandpaternal breed is associated with an increase in nitrogen excretion through influencing the underlying causes of variation in nitrogen excretion. In addition, no QTL for feed efficiency has been reported in this region of the genome; however, a QTL for ADG was reported in this region with an additive effect which is associated with growth [23]. Yue et al. [33] also reported a QTL for feed intake around this region.

During last stage of growth (120 to 140 kg), the QTL region on SSC2 associated with an increase in NEWG and TNE (115–116 cM) was not associated with any other traits in the current study. Although, QTL for ADG have been reported around this region [34], no QTL for feed intake or feed efficiency have been reported. This suggests that this region may be associated with underlying causes of variation in nitrogen excretion such as metabolism, protein utilisation, or maintenance requirements, etc.

During the entire analysed growing period, the QTL on SSC9 associated with nitrogen excretions, in the region between S0019 and SW2093, also was associated with an increase in ADEI, ADFI, APD and ADG at the last stage of growth (120 to 140 kg). This suggests the presence of a QTL for production, and in particular lean production, which increases feed intake and energy usage, and consequently results in an increased nitrogen excretion. Furthermore, the QTL associated with production shows a positive correlation with the QTL for feed intake which is in agreement with the genetic correlation between these traits [2]. Duthie et al. [8] reported QTL for ADG, APD, and ALD in this region using the same experimental data as in the current study.

## Conclusions

This study revealed six novel QTL for REI revealing the genomic architecture of efficiency in feed utilisation and indicating that the regulation of feed efficiency is partly independent from that of production traits. As expected no QTL for REI were overlapping with QTL for APD and ALD within the considered growth period, but between growth periods some overlapping occurred, suggesting change genomic regulations of the these traits during growth. One of the six novel QTL for REI had positional association with QTL for feed intake suggesting that some of the variation in REI can be explained by variation in feed intake. However, four of the six novel QTL for REI had positional association with QTL related to nitrogen excretions suggests the change in efficiency of feed utilisation due to underlying causes of variation in REI such as metabolism, digestion, protein turnover, etc. Different QTL for REI were identified at different growth stages, suggesting different genes are responsible for efficiency in feed utilisation at different stages of growth. This also suggests that selection for REI is most efficient if carried out within stages of growth.

## Competing interests

The authors declare they have no competing interests.

## Authors’ contributions

RR and PWK designed the experiment. MS, CAD and RR conceptualised the analysis. MS did quality control for the data and performed the analysis. ADW, EK and JAMVA contributed to the interpretation of the results and helped to draft the manuscript. All authors read, contributed, and approved the manuscript.
